# Molecular and Cellular Impact of Inflammatory Extracellular Vesicles (EVs) Derived from M1 and M2 Macrophages on Neural Action Potentials

**DOI:** 10.3390/brainsci10070424

**Published:** 2020-07-03

**Authors:** Sarah Vakili, Taha Mohseni Ahooyi, Shadan S. Yarandi, Martina Donadoni, Jay Rappaport, Ilker K. Sariyer

**Affiliations:** 1 Department of Neuroscience and Center for Neurovirology, Temple University Lewis Katz School of Medicine, Philadelphia, PA 19140, USA; tuf14559@temple.edu (S.V.); tug76893@temple.edu (T.M.A.); tuf33472@temple.edu (S.S.Y.); tug36939@temple.edu (M.D.); 2 Tulane National Primate Research Center, New Orleans, Covington, LA 70433, USA; jrappaport@tulane.edu

**Keywords:** M1and M2 macrophages, CD163, exosomes, action potential, neuroinflammation

## Abstract

Several factors can contribute to neuroinflammatory disorders, such as cytokine and chemokines that are produced and released from peripherally derived immune cells or from locally activated cells such as microglia and perivascular macrophages in the brain. The primary function of these cells is to clear inflammation; however, following inflammation, circulating monocytes are recruited to the central nervous system (CNS). Monocyte-derived macrophages in the CNS play pivotal roles in mediating neuroinflammatory responses. Macrophages are heterogeneous both in normal and in pathological conditions due to their plasticity, and they are classified in two main subsets, classically activated (M1) or alternatively activated (M2). There is accumulating evidence suggesting that extracellular vesicles (EVs) released from activated immune cells may play crucial roles in mediating inflammation. However, a possible role of EVs released from immune cells such as M1 and M2 macrophages on neuronal functions in the brain is not known. In order to investigate the molecular and cellular impacts of macrophages and EVs released from macrophage subtypes on neuronal functions, we used a recently established in vitro M1 and M2 macrophage culture model and isolated and characterized EVs from these macrophage subtypes, treated primary neurons with M1 or M2 EVs, and analyzed the extracellular action potentials of neurons with microelectrode array studies (MEA). Our results introduce evidence on the interfering role of inflammatory EVs released from macrophages in interneuronal signal transmission processes, with implications in the pathogenesis of neuroinflammatory diseases induced by a variety of inflammatory insults.

## 1. Introduction

Monocytes and macrophages have critical roles in inflammation and the pathogenesis of neuroinflammatory diseases [1,2]. Even though they originate from a common myeloid progenitor cell in the bone marrow, macrophages are functionally heterogeneous [3]. Macrophages induce an immune response by the phagocytosis of microorganisms [4]. They can also regulate local immune responses by producing cytokines/chemokines and other types of immune mediators. These cells range from resting residents to activated inflammatory macrophages that depend on the tissue sites and their activation status [1,5]. They are capable of expressing scavenger receptors, adhesion molecules, and receptors for soluble mediators, including cytokines, chemokines, and growth factors [3,5]. The tissue localization and activation status of macrophages lead to the expression of different receptors with a variety of functions [5]. Intracellular signal pathways in macrophages are often hard to study due to the fact that more than one of these receptors are involved in the biding of ligands [6]. Scavenger receptors belong to the group of pattern recognition receptors and are known for their broad range of ligand binding [7]. The scavenger receptors cysteine-rich (SRCR) is a family of structurally related transmembrane glycoproteins. There are two variants of the SRCR domain, which divide the SRCR molecules into two groups: group A and group B [8]. CD163 is a member of the SRCR family in group B [9,10]. Mature tissue macrophages expressing high levels of CD163 includes Kupffer cells in the liver, red pulp macrophages in the spleen, cortical macrophages of the thymus, resident mature bone marrow macrophages, perivascular and meningeal macrophages of the central nervous tissue, and scattered macrophages in various other tissues [11]. CD163-positive macrophages are found during the healing phase of acute inflammation and in chronic inflammation, whereas freshly infiltrated macrophages are usually negative for CD163 [12,13]. This suggests that CD163-positive macrophages may play a role in the resolution of inflammation [1,14]. CD163 expression can be modified by a variety of anti-inflammatory mediators, such as interleukin (IL)-10 and IL-6, and proinflammatory mediators like LPS, IFN, and TNF [15]. The regulation of CD163 by pro- and anti-inflammatory factors illustrated a link between CD163 immune suppression and the resolution of inflammation [8,16,17,18].

Cells exchange information through the secretion of soluble factors or through direct cell–cell interactions [8]. Cells release a variety of extracellular vesicles (EVs) into the environment that represent an important mode of intercellular communication due to carrying and delivering various cargos, including cytosolic proteins, growth factors, cytokines/chemokines, lipids, and RNA, that can be transferred to recipient cells [18,19]. EVs are classified based on their cellular origin and biological function or based on their biogenesis. These vesicles are 30-1000-nm in diameter and are released by a variety of cells, including immune cells, in which they may act as antigen-presenting vesicles, stimulants of antihumoral immune responses, or inducers of tolerogenic effects to suppress inflammation [19,20]. Extracellular vesicles are classified as exosomes, microvesicles (MVs), and apoptotic bodies. EVs are involved in many physiological processes, and they may have important roles in immune regulation for both nonimmune and immune cells [21,22,23]. In neuroinflammatory diseases, macrophages/microglia-derived EVs are shown to be involved in the propagation of inflammatory signals and cell-to-cell communications [23,24]. However, the possible role of EVs released by different subsets of macrophages (M1 and M2) in neuroinflammation and neuroinflammation-derived neurotoxicity triggered by a variety of pathogenic conditions are not known. We recently developed and described an in vitro M1 and M2 macrophage culture model [16]. Here, we further improved this model and isolated/characterized EVs from these macrophage subtypes. Primary neurons were treated with M0 (undifferentiated monocytes), M1, or M2 EVs and analyzed for their extracellular action potentials through a set of microelectrode array studies (MEA). Our results suggest that both M1 and M2 macrophages may possess neurotoxic effects mediated by EVs released by these cells in the context of neuroinflammation. These results, for the first time, revealed a novel neuronal insult mediated by inflammatory EVs released from macrophages that was not recognized before.

## 2. Materials and Methods

### 2.1. MonoMac-1 Cells and Induction of Cellular Differentiation into M1 and M2 Phenotypes 

**Figure 1:**
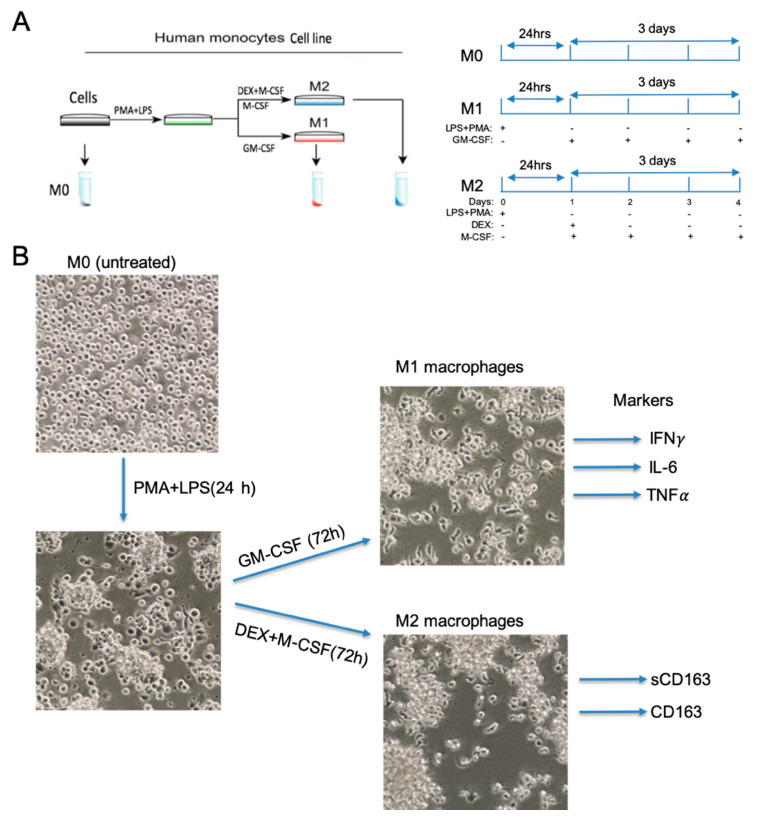
Differentiation of MonoMac-1 cells into functional M1 and M2 macrophage phenotypes. (**A**) Schematic presentation of MonoMac-1 differentiation and growth conditions of cells. MonoMac-1 cells (untreated M0) were first activated by phorbol 12-myristate 13-acetate (PMA) (20 ng/mL) and LPS (10 ng/mL) treatment for 24 h. The cells were then treated with granulocyte-macrophage colony-stimulating factor (GM-CSF) (10 ng/mL) for 72 h to differentiate into M1 type macrophages. For the M2 phenotype macrophages, cells were treated with macrophage colony-stimulating factor (M-CSF) (5 ng/mL) and dexamethasone (DEX) (200 ng/mL) for 72 h. (**B**) Morphological changes of macrophages with differentiation. Phase contrast images were taken at 20X magnification. M1 (IFN-γ, interleukin (Il)-6, TNF-α) and M2 (CD163 and sCD163) macrophage markers are shown.

MonoMac-1 cells (M0) were acquired from DSM (#ACC 252, Braunschweig, Germany). The MonoMac-1 cell line was initially derived from the Mono-Mac parental cell line, which was itself established from the peripheral blood of a patient diagnosed with an acute peripheral monoblastic leukemia [25,26]. According to morphological, cytochemical, and immunological traits, this cell line was assigned to the monocytic lineage [15,27]. Cells were cultured in RPMI 1640 medium supplemented with 10% heat-inactivated fetal bovine serum (#R8758, Sigma-Aldrich, MO, USA), 1% stable glutamine, 1-mM sodium pyruvic acid, and minimum essential medium nonessential amino acids (Sigma-Aldrich, MO, USA). Cell were maintained in a humidified chamber kept at 37 °C and 5% CO_2_. Cells were seeded at a density of 0.5 × 10^6^ cells/mL and subcultured to 1 × 10^6^ cells/mL, with a complete media change every 2 or 3 days. To promote macrophage-like differentiation, MonoMac-1 cells were seeded at a density of 1 × 10^6^ cells/mL in 100-mm dishes and cultured in the same media supplemented with 20-ng/mL phorbol-12-myristate-13-acetate (#1201/1, R&D Systems, MN, USA). Furthermore, half of the cells were incubated with 10-ng/mL lipopolysaccharide (#L2630, Sigma-Aldrich, MO, USA) for 24 h. Afterwards, cells were washed twice in phosphate-buffered saline (PBS) and differentiated to M1 and M2 macrophage phenotypes [15]. Cells incubated in a culture media containing 5-ng/mL granulocyte-macrophage colony-stimulating factor (GM-CSF) (#215-GM-010, R&D Systems, MN, USA) to differentiate into M1 phenotype macrophages for 3 days (Figure 1). Regarding the M2 phenotype, cells were treated with 5-ng/mL macrophage colony-stimulating factor (M-CSF) (#216-MC-005, R&D Systems, MN, USA) and 200-ng/mL dexamethasone (DEX) (#1126/100, R&D Systems, MN, USA) daily (Figure 1).

### 2.2. Purification and ZetaView Analysis of Extracellular Vesicles (EVs) from MonoMac-1 (M0) and M1 and M2 Cells 

Culture media of undifferentiated MonoMac-1 (M0) or differentiated M1 and M2 cells were obtained from 1 × 10^7^ cells of each cell types. EVs were purified from these supernatants utilizing the differential centrifugation method. Approximately 10-mL growth media from each cell type was first centrifuged at 3000× *g* for 30 min at 4 °C (Eppendorf Centrifuge, 5804R) to clear cell debris followed by a centrifugation at 10,000× *g* for 30 min at 4 °C (HB-6 rotor, Sorval Centrifuge, RC6+, Thermo Scientific), followed by filtration (Corning Incorp., NY, USA). At this step, clear supernatants were either stored at 4 °C or proceeded for ultracentrifugation. Ultracentrifugation was performed at 100,000× *g* for 4 h in a Beckman Ultracentrifuge. After centrifugation, the tubes were inverted to remove the remaining liquid and washed with PBS. The EV pellets were resuspended in 200-ul PBS. Regarding the zeta view analysis, EVs were diluted (1:250) in PBS to a final volume of 2 mL. For each measurement, three cycles were performed by scanning 11 cell positions each and capturing 60 frames per position (video setting: high) after capture; the videos were analyzed by the in-build Zeta View Software 8.02.31 with specific analysis parameters: maximum particle size: 1000, minimum particle size 5, and minimum particle brightness: 20. Hardware: embedded laser: 40 mW at 488 nm and camera: CMOS.

### 2.3. ELISA (Enzyme-Linked Immunosorbent Assay) 

All ELISA assays were performed based on instructions provided by the manufacturer (R&D system, MN, USA). Culture media of the cells were centrifuged at 450× *g* for 5 min. Supernatants were collected and analyzed for IL-6 (#D6050), CD163 (#DC1630), TNF-alpha (#DTA00C), and IFN-gamma (#DIF50) levels.

### 2.4. Multielectrode Array (MEA) Recordings

MEA recording was performed in the MEA-1060 system (#10iR-ITO-gr, Multichannel Systems, Reutlingen, BW, Germany), providing 60 simultaneous recordings from each condition. Each array contains 60 titanium nitride (TiN) electrodes covering a rectangular grid. Each electrode is composed of a circular TiN pad of a 30-μm diameter, where the array spacing between every two neighboring electrodes is 100 μm. First, the MEAs underwent sterilization via applying 70% ethanol and then exposing the arrays to UV light for 30 min. As the MEA surface is originally hydrophobic, poly-D-lysine was used to hydrophilize the MEAs, as well as to provide a layer to enhance the cell adhesion to the MEAs, and poly-D-lysine (P6407, Sigma-Aldrich, MO, USA) was diluted in PBS with a final concentration of 1 mg/mL and applied to the MEA surface for 2 h at 37 °C. Subsequently, laminin (#23017015, Invitrogen/Thermo Fisher, Inc., Waltham, MA, USA) was coated onto MEAs (overnight at 37 °C) to support long-lasting cellular adhesion (for > 10 day cell cultures) and to improve the neural processes’ development. Once the MEAs were sterilized, primary embryonic rat neurons (PERNs derived from the hippocampi of E18 rat embryos) were plated on them, with the average density of one million cells per MEA (1 × 10e^6^ cells/well). Neurons are required to stay and develop processes on the MEA for at least 25 days before the treatments start. At this age, neurons exhibit basal simultaneous firing and synchronous firing across the MEA. During this period, neurons were maintained regularly using a specialized serum-free medium, and their activity was monitored periodically. After neurons reached acceptable basal activity [28,29], experimental recordings were started before the EVs treatment (0 h), and immediately, the cells were treated by extracellular vesicles (EVS) isolated from M0, M1, and M2 cells (500 EVs/cell). Recordings were repeated at 0, 8, 24, and 48 h posttreatments. The recorded data were transferred to the MATLAB environment to be processed using custom-developed algorithms and codes. To decrease the amount of noise, an offline band-stop filter was applied to the recorded datasets. The filtered data were then employed to estimate the neuronal firing amplitude and frequency under all experimental conditions. Peak detection algorithm was used to determine the number of firings per unit time exceeding three standard deviations of the data.

### 2.5. Flow Cytometry Analyses 

Flow cytometric analyses were carried out on nontreated and treated MonoMac-1, using fluorochrome-conjugated antibodies, CD163-R-PE (#333605, Mac2-158; Trillium Diagnostics, ME, USA). The antibody-fluorochrome panel was designed according to the optimized detection. Isotype controls were utilized in the construction of the panel in order to ensure the specificity of each antibody. Fluorescence minus one (FMO) and cells incubated without antibodies were used to assist in gating positive versus negative populations. Thirty thousand events per test were collected on a 4-laser, 13-color LSRII Analyzer (BD) and analyzed using the FlowJo, (version 10.1r5; TreeStar, Ashland). The Live/Dead Fixable Blue Dead Cell Stain Kit (Invitrogen, Grand Island) was used to exclude nonviable cells from analysis.

### 2.6. Statistical Analyses

All frequencies of data were compared using one-way repeated measures ANOVA with Tukey’s post-hoc test. Statistical analyses were performed using GraphPad Prism (version 6) software. *p*-values ≤ 0.05 were statistically significant.

## 3. Results

### 3.1. Generation and Characterization of M1 and M2 Macrophages from MonoMac-1 Cells

Differentiation of monocytic cell lines with phorbol 12-myristate 13-acetate (PMA) is widely used as a model for studying the function and biology of human macrophages [30,31]. We have previously used a cell line derived from an acute myelogenous leukemia (AML) cell line, MonoMac-1, and established conditions to promote the expression of specific macrophage markers [15]. These conditions involved the glucocorticoid pathways and cFMS signaling based on the action of dexamethasone and macrophage colony stimulating factor (MCSF) in promoting macrophage differentiation, in addition to the PMA and LPS treatments [15]. By improving our previously established methodology, here, we utilized MonoMac-1 cells for the generation of macrophages with distinct phenotypes. MonoMac-1 cells were initially derived from the MonoMac parental cell line, which was itself established from the peripheral blood of a patient diagnosed with an acute peripheral monoblastic leukemia [25,26]. According to morphological, cytochemical, and immunological characterizations, this cell line was assigned to the monocytic lineage. Since both the PMA and LPS treatment mediates monocyte differentiation into macrophages [15,31,32], we started by incubating MonoMac-1 cells with PMA (20 ng/mL) and LPS (10 ng/mL) for 24 h (Figure 1A). As assessed by light microscopy, after 24 h of post-differentiation, MonoMac-1 cells became larger, more flattened and became adherent to culture dishes (Figure 1B). In order to differentiate the cells into classical macrophages (M1), granulocyte-macrophage colony-stimulating factor (GM-CSF, 10 ng/mL), the main cytokine associated with M1 activation [30], was added daily for 72 h (Figure 1A,B). On the other hand, cells were treated with macrophage colony-stimulating factor (M-CSF) alone and combined with dexamethasone (DEX) for 72 h to differentiate into M2 macrophages (Figure 1A,B). M1 and M2 polarized macrophages were indistinguishable by their morphology (Figure 1B).

**Figure 2:**
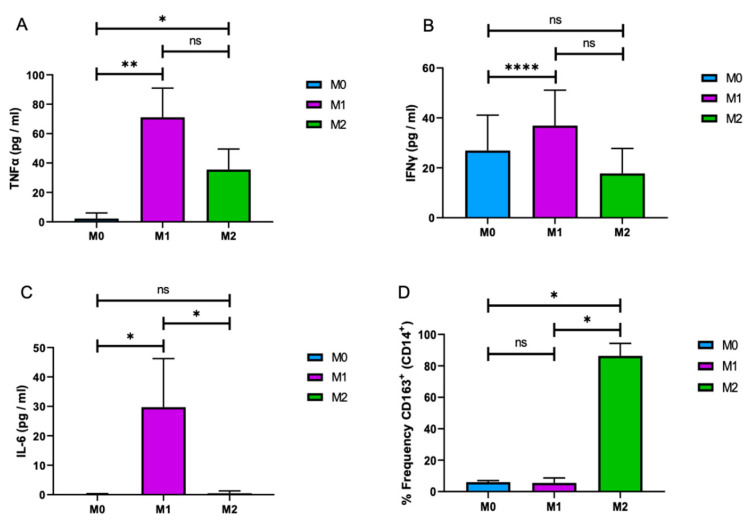
Differential cytokine expression of M1 and M2 macrophages. M1 and M2 macrophages were differentiated from MonoMac-1 cells (M0), and growth media of the cells was subjected to the Enzyme-linked Immunosorbent Assay (ELISA) for TNF-α (**A**), IFN-γ (**B**), and IL-6 (**C**). (**D**) M0, M1, and M2 cells were analyzed by flow cytometry for the frequency of CD163+ and CD14+ macrophages. (*n* = 3; * *p* ≤ 0.05, ** *p* ≤ 0.01, and **** *p* ≤ 0.001).

M1 and M2 macrophages can be characterized and differentiated based on the expression of proinflammatory and inflammatory cytokines/chemokines [8,33]. Here, we further characterized M1 and M2 macrophages by the expression of macrophages markers TNF-α, IFN-γ, IL-6, and sCD163. Cells were differentiated as described above, and culture media were subjected to ELISA, as described in Materials and Methods. As shown in Figure 2A, the levels of TNF-α, IFN-γ, and IL-6 were significantly higher in cultures of M1 macrophages than those in M0 and M2. Among these cytokines, IL-6, a well-characterized M1 cytokine [33], showed a robust M1 expression than the others that validated the differentiation of MonoMac-1 cells into functional M1 macrophages (Figure 2C). On the other hand, CD163, a well-defined marker of M2 macrophages, was used as a marker to assess the M2 macrophage differentiation. Flow cytometry analysis revealed that, while 90% of M2 macrophages were positive for CD163, the frequency of CD163-positive M0 and M1 cells was less than 5% (Figure 2D). These results suggest that MonoMac-1 cells can be differentiated into the functional M1 and M2 macrophages with the defined methodology and be utilized as a tool to study the distinct functions of these cells in culture conditions.

### 3.2. Isolation and Characterization of Extracellular Vesicles (EVs) Released from M1 and M2 Macrophages

**Figure 3:**
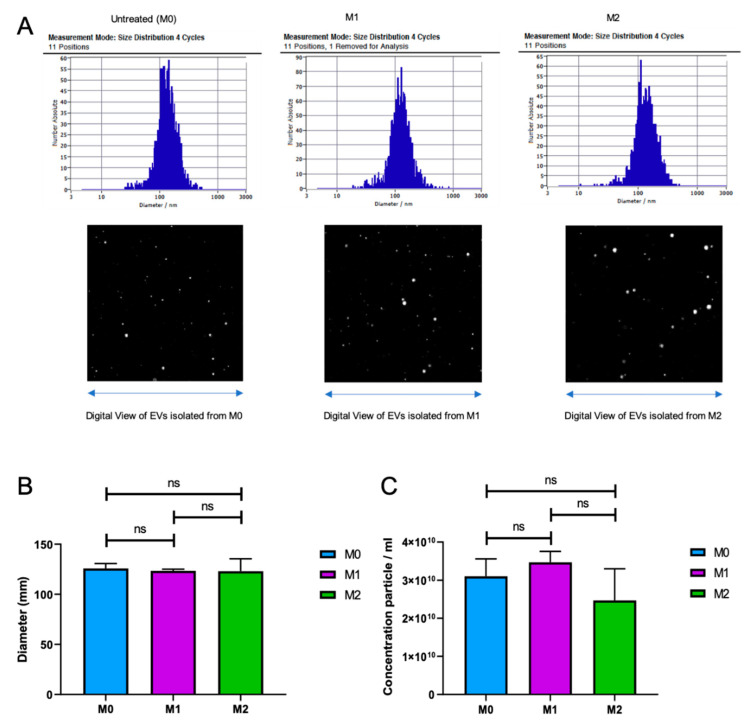
Extracellular vesicles (EVs) isolated from M0, M1, and M2 cells are indistinguishable. (**A**) Zeta view analysis of the EVs extracted from M0, M1, and M2 macrophages. Size distribution in 4 cycles—11 positions and digital view of EVs from each cell type are shown. The size (diameter) (**B**) and concentration (particle number) (**C**) of EVs extracted from M0, M1, and M2 macrophages are shown as bar graphs. (*n* = 3).

There is accumulating evidence suggesting that extracellular vesicles (EVs) released from activated immune cells may play crucial roles in mediating inflammation [23,24]. However, a possible role of EVs released from immune cells such as M1 and M2 macrophages on neuronal functions in the brain is not known. Therefore, here, we first isolated and characterized EVs from M1 and M2 macrophage subtypes. M1 and M2 macrophages were differentiated from MonoMac-1 cells, as described above. Cells were grown in complementary growth media containing 10% EV-depleted FBS to avoid EV contamination from bovine serum. EVs were isolated from these cultures by differential centrifugation, as described in Materials and Methods. The purified EVs were subjected to zeta view nanoparticle tracing and tracking analysis. As shown in Figure 3A,B, the average size of the EVs released from M0, M1, and M2 cells were around 125 nm, with no significant variation between cell types. In addition, although a noticeable decrease was observed for M2 macrophages, there were no significant variations in the number of EV particles released by all three cell types (Figure 3C). These results suggest that M0, M1, and M2 cells generate and release EVs that are indistinguishable in their sizes and concentrations.

### 3.3. CD163 is Released from M2 Macrophages in Association with EVs

**Figure 4:**
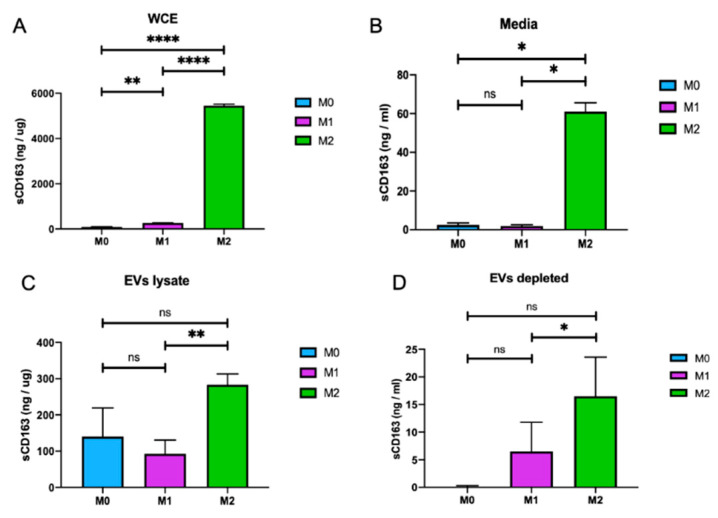
Soluble CD163 (sCD163) is associated with M2-EVs. (**A**) The level of CD163 in whole-cell protein extracts (WCE) from M0, M1, and M2 cells was measured by ELISA and shown as a bar graph. (**B**) Soluble CD163 levels in the growth media of cells from M0, M1, and M2 cells were analyzed by ELISA and shown as a bar graph. (**C**) sCD163 levels in EV lysates isolated from M0, M1, and M2 cells were analyzed by ELISA and shown as a bar graph. (**D**) Soluble CD163 levels in the growth media of cells were analyzed by ELISA after EV isolation (EV-depleted, the remaining growth media of cells from panels B and C after EV isolations). (*n* = 3; * *p* ≤ 0.05, ** *p* ≤ 0.01, and **** *p* ≤ 0.001).

Here, we sought to further explore the expression of CD163 by M2 macrophages and its possible association with EVs released by these cells. M0, M1, and M2 macrophages were cultured, and growth media of the cells was processed for EV isolation, as described above. The growth media of the cells were recovered after EV isolation and named EVs-depleted media. Whole-cell protein lysates were also prepared from cells in the same experimental settings. sCD163 levels were measured by CD163 ELISA assay in whole-cell protein lysates, in growth media of the cells, in lysates from EVs isolated from the growth media and in EV-depleted growth media of the cells. As expected, there was a significant increase in CD163 expression levels in whole-cell protein extracts isolated from M2 macrophages (5500 ng/mg) and in the growth media of these cells (60 ng/mL) compared to M0 and M1 cells (Figure 4A,B, respectively). To further investigate the possible association of CD163 with EVs isolated from M2 macrophages, CD163 levels in EVs lysates and EVs-depleted media of M0, M1, and M2 cells were also analyzed. Interestingly, the level of CD163 in EV lysates from M2 macrophages (300 ng/mg) was significantly higher than M0 and M1 macrophages (Figure 4C). More interestingly, the level of sCD163 in EV-depleted media from M2 macrophages was decreased to 17 ng/mL from 60 ng/mL (compare Figure 4D with Figure 4B), suggesting that a major amount of sCD163 was associated with EVs released by M2 macrophages. In conclusion, our data further confirms that CD163 is a M2 macrophage marker. In addition, these observations suggest for the first time that CD163 is released from M2-like macrophages into the extracellular matrix in association with EVs.

### 3.4. Effect of Extracellular Vesicles (EVs) Released from M0, M1, and M2 Macrophages on Neuronal Electrophysiological Activity 

**Figure 5:**
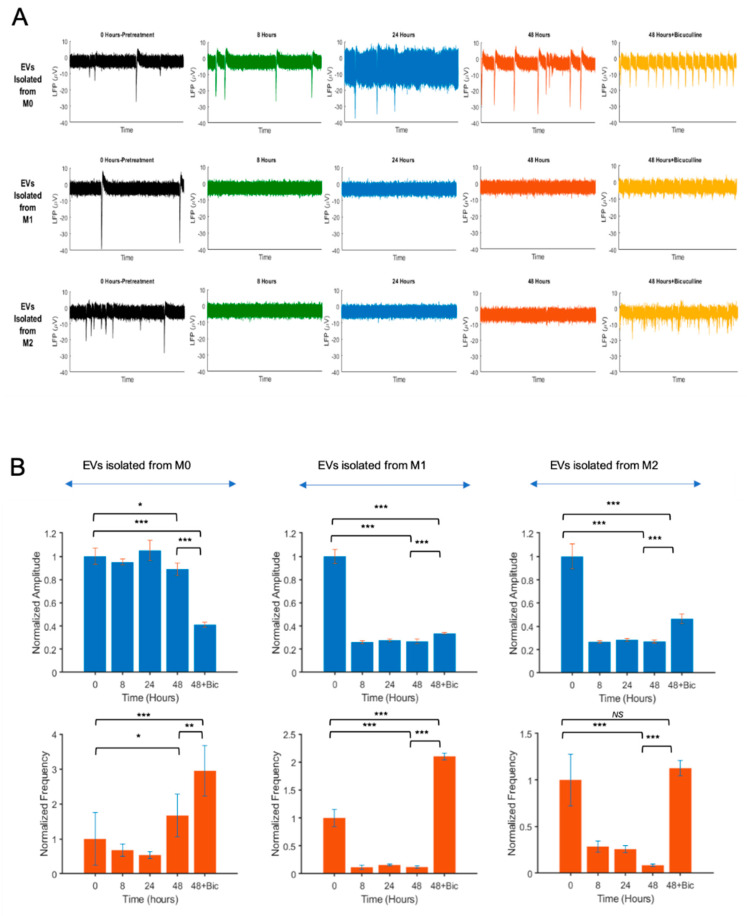
Effect of EVs isolated from M0, M1, and M2 on neuronal activity by microelectrode array studies (MEA). (**A**) Extracellular action potential recordings of primary rat neurons treated with EVs from M0, M1, and M2 macrophages. Rat hippocampal neurons were dissociated from the hippocampi of E18 prenatal rat embryos and placed onto the MEA dishes. Experimental recordings and EV treatments were started when the cultures were 21 days old. After placing the MEAs on the amplifier, recordings were performed using the MC_Rack software. The recorded electrophysiological activities are shown for a 60-s duration of the recordings. The recorded spiking data (in μV versus time) were transferred to the MATLAB environment for further offline analysis. Top row: M0-Evs at *t* = 0, 8, 24, and 48 h. Middle row: M1-EVs treated neurons at *t* = 0, 8, 24, and 48 h. Bottom row: M2-EVs treated neurons at *t* = 0, 8, 24, and 48 h. At 48 h posttreatments, cells were also treated with bicuculline and recorded. (**B**) Time-dependent variations of the normalized firing frequency and amplitude of cells treated with EVs isolated from M0, M1, and M2 macrophages are shown as bar graphs. (*n* = 3; * *p* ≤ 0.05, ** *p* ≤ 0.01, and *** *p* ≤ 0.001).

To examine the effect of extracellular vesicles (EVs) isolated from M0, M1, and M2 macrophages on the neuronal electrical activity, we employed microelectrode array (MEA) technology that provides a high-throughput, simultaneous, and noninvasive platform to record neuronal extracellular action potentials from multiple sites. Primary embryonic rat neurons (PERNs) were cultured in MEA dishes, as described in the Materials and Methods. EVs were purified from culture supernatants of M0, M1, and M2 macrophages and analyzed/characterized by zeta view, as described in Figure 3. PERNs were treated with M0, M1, and M2 EVs (500-EV particles per cell). MEA extracellular action potential recordings were performed at 0 (pretreatment), 8, 24, and 48 h posttreatments. The recorded electrophysiological activities are shown for a 60-s duration of recordings. As illustrated in Figure 5A, the action potentials of neurons treated with EVs isolated from M1 and M2 macrophages were dramatically suppressed at 8 h posttreatments, with no significant recovery at 24 and 48 h. To further investigate the effect of EVs isolated from different macrophage phenotypes on neuronal activity, we analyzed the firing rate of the neural spikes within 48 h posttreatments. Interestingly, M1 and M2 EVs dramatically reduced the spike frequency, as compared to M0 EVs (Figure 5B). Moreover, the signal amplitudes of neuronal firing were also reduced by M1 and M2 EVs in parallel to the spike frequencies (Figure 5B). 

We hypothesized that the reduced activity of the action potential could be due to the increased activity of inhibitory receptors of the neurons induced by M1 and M2 EVs. To examine this hypothesis, and also to show that the loss of activity was not due to the synapses loss or neuronal death, we utilized bicuculine, an antagonist of inotropic GABA_A_ receptors, which constitutes major inhibitory receptors in hippocampus. Neurons were treated with bicuculline (25 μM) at 48 h posttreatments. Immediately after bicuculline application, the neurons treated with M1 and M2 EVs produced medium-to-high amplitude bursts compared to the prebicuculline treatments (Figure 5B). In both M1 and M2 EV-treated neuronal cultures, bicuculline led to a significant increase in the spike frequencies, suggesting that the reduced action potential induced by EVs from M1 and M2 macrophages is reversible by GABA_A_ receptor antagonists. 

**Figure 6:**
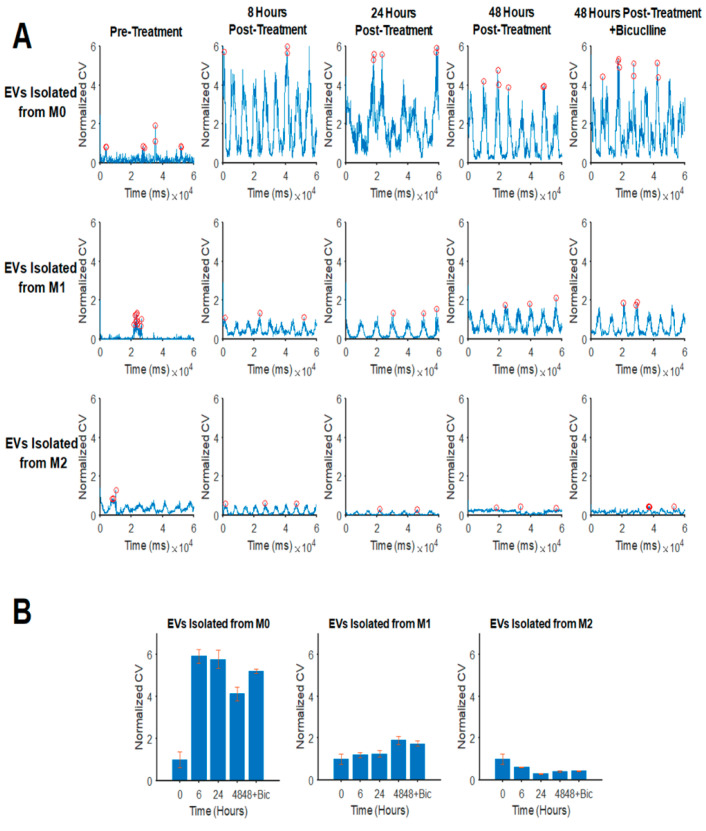
Effect of EVs isolated from M0, M1, and M2 on the neuronal conduction velocity (CV). (**A**) EVs isolated from M1 and M2 cells inhibit neuronal CVs. Conduction velocities were calculated and quantified using 60 simultaneous recordings. As depicted here, the maximum CV decreased upon treatment with M2-derived EVs, as opposed to M1-derived and M0-derived EVs. Bicuculline was added to neurons at 48 h posttreatment for 20 min to determine whether the M2-EV-induced loss of conduction velocity could be (partially) restored as the GABA _A_ receptors are inhibited. (**B**) The quantifications of the peak conduction velocities for the experimental conditions indicate the adverse effect of M1- and M2-derived EVs on neuronal CVs over the course of 48 h posttreatments. (*n* = 3).

We also studied the impact of EVs released from M1 and M2 macrophages on the neuronal networks, as revealed by network analysis-derived conduction velocities (CV) of the wave propagation (Figure 6A,B). Further details regarding the technical aspects of the MEA data analysis, including the CV, were previously reported by us [28]. Interestingly, EVs from M0 cells resulted in a significant increase in CVs at 8 h posttreatments, and this effect was stable for up to 48h posttreatments. On the other hand, EVs from M1 macrophages had a minimal effect on CVs during 24 h, but it caused an increase in CVs at 48-h posttreatments. More interestingly, unlike M0 and M1, EVs isolated from M2 macrophages caused a decrease in the conduction velocities by approximately 50% compared to the pretreatments. The bicuculline treatment had no significant effect on the CV of neurons treated with EVs. 

## 4. Discussion

Monocytes and macrophages constitute an important component of immune responses against viruses, bridging innate and adaptive immunities [34,35]. Macrophage differentiation is correlated with a decrease in the nucleocytoplasmic ratio of monocytes due to a growth in cytoplasmic volume [31]. Macrophage heterogeneity is influenced by the differentiation state, with marked differences between monocytes and macrophages [31]. Commonly used protocols are used to induce macrophage differentiation from monocytic cell lines, but a derivation towards highly differentiated macrophage phenotypes has not been a major focus of prior studies. In this study, we identified a number of macrophage characteristics associated with differentiation, which vary in MonoMac-1 cells treated with different protocols. Our results showed that a protocol of treating MonoMac-1 cells with PMA and LPS followed by a period of further culturing with GM-CSF, M-CSF, and DEX drives cells towards differentiated macrophage phenotypes. Here, we demonstrated that PMA+LPS sets the stage for the cells into macrophage differentiation, and GM-CSF and M-CSF+DEX promote the expansion of the M1 and M2 macrophages, respectively. M1 and M2 macrophages are shown to express differential proinflammatory and inflammatory cytokines/chemokines [8,33]. Here, we further characterized our MonoMac-1-derived M1 and M2 macrophages by the expression of TNFα, IFN-γ, IL-6, and sCD163 (Figure 2). For the M2 phenotype, a flow cytometry analysis revealed that the cells were treated PMA+LPS+M-CSF, and DEX expressed the high level of CD163 (Figure 2D). Our results defined that MonoMac-1 cells can be differentiated into functional M1 and M2 macrophages and be utilized as a tool to study distinct functions of these cells in culture conditions. 

Extracellular vesicles (EVs) are potent vehicles of intracellular signaling, and cells transfer information through the secretion of these vesicles [36]. Extracellular vesicles have been isolated from bodily fluids, and they have a key role in both the regulation of normal physiological processes and the pathology underlying several diseases [37]. Quantitatively, we compared the size and concentration of EVs isolated from M0, M1, and M2 phenotypes via zeta view. Interestingly, the size and concentration of EVs isolated from these cells were indistinguishable. The average size of the EVs isolated from M0, M1, and M2 cells were around 125nm, with no significant changes between them. Furthermore, although there was a reduction in the number of EV particles for M2 macrophages, there were no significant variations between all three cell types (Figure 3). Moreover, for the first time, we illustrated the association of CD163 with EVs. Considerably, the level of sCD163 in EV lysates isolated from M2 macrophages (300 ng/µg) was much higher than those from M0 (140 ng/µg) and M1 (100ng/µg) cells (Figure 4). Since sCD163 is mainly expressed by M2 cells, these results suggest that CD163^+^ EVs may serve as a marker of M2-mediated inflammation. One can also argue that, although the size and concentration of EVs were similar, the content of EVs released by M1 and M2 phenotypes may vary, as suggested by the CD163 association with M2-EVs. On the other hand, the true contents of M1 and M2 EVs for proteomics, miRNA and mRNA profiles, and full profiles of cytokine/chemokine associations remain to be determined.

The functional impact of EVs released from M0, M1, and M2 cells on the neuronal electrophysiological activity was analyzed by microelectrode array (MEA) studies. Our MEA studies of hippocampal primary neurons showed that EVs isolated from M1 and M2 cells suppress network-wide hippocampal neuronal electrical activity. Several studies show that, in the brain, in addition to classical synaptic neurotransmission, neurons transfer information through the secretion of extracellular vesicles that can contribute to the range of neurobiological functions [38,39]. Here, as a first study of a network-wide activity assessment under macrophage neuropathogenesis, we utilized MEA analyses of hippocampal neurons in the presence of EVs isolated from macrophages. Using this technique, MEAs were used to record from thousands of neurons in a simultaneous, multisite, and noninvasive manner. Our results indicated an inhibitory effect of EVs isolated from macrophages on different aspects of neuronal firing, including decreasing the numbers of bursts, spikes per burst, and firing amplitude. The frequency of spikes and amplitudes in neurons decreased extremely in both EVs M1 and M2 treatments at 8 h posttreatments. The activity of the neurons was partially restored after treatment with bicuculline at 48 h post-EV treatments. This restoration was more apparent in the neuronal amplitude. Although the peak amplitude did not recover completely compared to the pretreatment conditions, our data indicate that EVs from M1 macrophages restore rather quickly in comparison with EVs from the M2 treatment. This observation may explain the stronger inhibitory and/or neurotoxic effect of M2-EVs on neuronal activity. The inhibition of GABA_A_, on the other hand, and to a lesser extent, altered the neuronal excitability in the presence of EVs isolated from M2 macrophages. Furthermore, the impact of EVs isolated from M1 and M2 macrophages on the neuronal networks was also analyzed by network analysis-derived conduction velocities (CV) of the wave propagation. While EVs isolated from M0 cells increased CV, M1, and M2, EVs did not show the same trend. These observations signify the greater negative impacts of M1 and M2-derived EVs on neuronal activities as compared to the M0 (control) group, and this effect is likely not being induced as a result of neuronal or synaptic loss but, rather, occurs due to the attenuation of neuronal activity partially mediated by the increased activity of inhibitory pathways in the hippocampus.

## 5. Conclusions

In conclusion, this study, for the first time, introduces evidence on the interfering role of inflammatory EVs released from macrophages in interneuronal signal transmission processes, with implications in the pathogenesis of neuroinflammatory diseases induced by a variety of insults.
